# Survival outcomes for neoadjuvant versus adjuvant chemotherapy in early breast cancer patients

**DOI:** 10.1093/oncolo/oyaf356

**Published:** 2025-11-18

**Authors:** Chengshi Wang, Jianhui Zhang, Juecai Chen, Xiaoyan Zhang, Songbo Zhang, Purong Zhang, Junjie Li

**Affiliations:** Department of Breast Surgery, Sichuan Clinical Research Center for Cancer, Sichuan Cancer Hospital & Institute, Sichuan Cancer Center, Affiliated Cancer Hospital of University of Electronic Science and Technology of China, Chengdu 610041, China; Department of Breast Surgery, Sichuan Clinical Research Center for Cancer, Sichuan Cancer Hospital & Institute, Sichuan Cancer Center, Affiliated Cancer Hospital of University of Electronic Science and Technology of China, Chengdu 610041, China; Department of Hematology, The First Affiliated Hospital of Chengdu Medical College, Chengdu 610500, China; Department of Emergency, West China Second University Hospital, Sichuan University, Chengdu 610041, China; Department of Breast Surgery, Sichuan Clinical Research Center for Cancer, Sichuan Cancer Hospital & Institute, Sichuan Cancer Center, Affiliated Cancer Hospital of University of Electronic Science and Technology of China, Chengdu 610041, China; Department of Breast Surgery, Sichuan Clinical Research Center for Cancer, Sichuan Cancer Hospital & Institute, Sichuan Cancer Center, Affiliated Cancer Hospital of University of Electronic Science and Technology of China, Chengdu 610041, China; Department of Breast Surgery, Sichuan Clinical Research Center for Cancer, Sichuan Cancer Hospital & Institute, Sichuan Cancer Center, Affiliated Cancer Hospital of University of Electronic Science and Technology of China, Chengdu 610041, China

**Keywords:** neoadjuvant chemotherapy, adjuvant chemotherapy, breast cancer, prognosis, survival

## Abstract

**Background:**

Neoadjuvant chemotherapy (NACT) has been widely used in breast cancer patients. The aim of the study was to compare survival outcomes between breast cancer patients receiving NACT, with and without complete pathologic response (pCR), and those receiving adjuvant chemotherapy (ACT).

**Methods:**

Based on the Surveillance, Epidemiology, and End Results database, we conducted a population-based cohort study including 48 350 breast cancer patients, 15 525 of whom with pCR after NACT, and 124 202 patients after ACT during the period of 2010-2021. In comparison with patients in ACT group, we assessed hazard ratios (HRs) of breast cancer-specific and overall mortality among individuals in NACT using Cox regression.

**Results:**

During the period of follow-up (median 5 years), 4800 and 8257 breast cancer–related deaths were identified among patients in NACT and ACT group, respectively. Patients in NACT group had unfavorable molecular type (human epidermal growth factor receptor 2 overexpression, triple negative), more advanced tumor features (higher grade and stage) and was more likely to undergo mastectomy and radiotherapy. Moreover, patients undergoing NACT had higher cumulative mortality rate of breast cancer (19.60% vs 10.46%), compared with those receiving ACT. After controlling for covariates, NACT patients were at increased risk of breast cancer-specific mortality (HR 1.47, 95% CI 1.41-1.53) compared with ACT patients. In contrast, NACT patients with pCR were associated with an improved breast cancer-specific survival (HR 0.59, 95% CI 0.54-0.64). The elevated risk was obviously greater among NACT patients in NACT-disfavored subgroups including lobular/mixed histology, well/moderately differentiated grade, local cancer stage, or HR+/HER2- molecular subtype (HRs 1.63-1.93).

**Conclusions:**

NACT patients have worse survival, compared with their ACT counterparts. Although patients with pCR after NACT derive significant survival benefits, NACT-disfavored subgroups may gain limited benefit from NACT, and alternative approaches should be considered.

Implications for Practice Breast cancer patients who achieve pCR following NACT show significantly improved survival rates, particularly in certain subtypes. Conversely, NACT patients with non-pCR or those from NACT-disfavored subgroups including lobular/mixed histology, well/moderately differentiated grade, local cancer stage, or HR+/HER2- molecular subtype, show limited survival benefits. Our analysis emphasizes the need for more personalized treatment strategies.

## Introduction

Breast cancer remains the most commonly diagnosed malignancy and the second leading cause of cancer-related mortality among women in the United States. In 2025, approximately 316 950 American women were diagnosed with breast cancer, and an estimated 42 170 women died of the disease.^1^ Over the decades, the treatment landscape for early-stage breast cancer has evolved significantly, with a major shift from adjuvant chemotherapy (ACT) to neoadjuvant chemotherapy (NACT) for certain subgroups of patients. Traditionally, ACT has been the standard of care following surgery, but recent advances have highlighted the potential benefits of NACT, particularly in locally advanced, triple-negative breast cancer (TNBC) and human epidermal growth factor receptor 2 overexpression (HER2+) patients.^2^^,^^3^ NACT offers the advantage of assessing treatment response in real-time and can not only downstage for breast conservation^4^^,^^5^ but also de-escalate axillary surgery,^6^ potentially showing similar survival outcomes (eg, disease-free survival, breast cancer survival, and overall survival) as ACT.^5^^,^^7^^,^^8^ Additionally, NACT provides an opportunity to evaluate molecular biomarkers and residual disease after treatment, which has become a key factor in understanding prognosis. Recent findings of 12 neoadjuvant trials showed that pathological complete response (pCR), defined as no residual invasive tumor in the breast or lymph nodes, was significantly associated with event-free survival and overall survival.^9^ Some studies have suggested patients with aggressive tumor types, such as TNBC and HER2-positive (HER2+) tumors, tended to benefit more from NACT due to higher pCR rates, which are associated with improved survival outcomes.^9–11^ Conversely, some studies have indicated that NACT patients with hormone receptor positive (HR+)/HER2-negative (HER2-) or lobular histology often exhibit a lower total pCR rate.^12^^,^^13^ This variability might be attributed to differences in tumor biology and the intensity of systemic therapy.^9^ Besides, KATHERINE,^14^ CREATE-X,^15^ monarchE,^16^ and OlympiA^17^^,^^18^ trials have suggested the perspective of postoperative treatment for NACT patients who had residual cancer burden with HER2+, TNBC, HR+/HER2-, and germline BRCA1/2 mutation diseases, respectively, leading to improved survival outcomes.^19^ Therefore, NACT has allowed a window of opportunity to conduct clinical trials and investigate additional biomarkers of molecular biology, developing new systemic approaches.^20–22^

Despite of the growing body of evidence supporting the survival benefit of NACT patients in certain subgroups, the survival effectiveness of NACT vs ACT remains debate, particularly in population-based analyses. We analyze data from the Surveillance, Epidemiology, and End Results Program (SEER) database to contribute to the evidence by comparing breast cancer-specific and overall mortality between NACT and ACT in a large cohort of early-stage breast cancer patients. Specifically, we assess the role of pCR in the neoadjuvant setting and its implications for survival outcomes, while also considering the evolving treatment paradigms for different molecular subtypes.

## Methods

### Study population

The SEER database contains information on demographic, tumor and clinical characteristics, and follow-up covering about 48% of the US population.^23^ Considering that the data for breast cancer patients treated with NACT was collected from 2010, we conducted a population-based cohort study of breast cancer patients diagnosed between January 1, 2010 and December 31, 2021 in the United States.

We first identified 528 519 patients undergoing NACT or ACT with pathologically confirmed early first primary breast cancer. We then excluded patients whose county was unknown (*N* = 747), those younger than 20 years old at the time of cancer diagnosis (*N* = 29), males (*N* = 3847), those with breast cancer in situ (*N* = 5620), stage IV (*N* = 27 880) or unknown stage (*N* = 21 731), and those whose information on surgery (*N* = 15 211) or chemotherapy (*N* = 280 902) was no/unknown. All patients were followed from breast cancer diagnosis until death, or December 31, 2021, whichever occurred first. In the end, 48 350 NACT patients (including 15 525 pCR, 19 998 non-pCR and 12 827 response to NACT but not noted if complete or partial response; Table S1) and 124 202 ACT patients were included for analysis. The selection flowchart was illustrated in Figure S1.

### Ascertainment of mortality

In SEER program, patients were followed-up by connecting registries in healthcare institutions. To ensure maximal follow-up, personal contacts were additionally considered for those who were regarded as having failed access. Breast cancer-specific and overall mortality were considered as the primary and secondary outcomes, respectively.

### Demographic, tumor, and clinical characteristics

We acquired information on year of diagnosis (2010-2013, 2014-2017, or 2018-2021), age at the year of cancer diagnosis, race (White, Black, Asian, or other), cohabitation status (non-cohabitation, cohabitation, or unknown), county (counties in metropolitan areas of larger than 1 million population, counties in metropolitan areas of 250 000 to 1 million population, counties in metropolitan areas of less than 250 thousand population, nonmetropolitan counties not adjacent to a metropolitan area, nonmetropolitan counties adjacent to a metropolitan area) and cost of living in family (low, middle, and high). We also obtained information on anatomic cancer stage (I, II, and III), histology (duct, lobular, mixed, or other), tumor grade (well, moderately, or poorly differentiated/undifferentiated, or unknown), status of estrogen receptor (ER), progesterone receptor (PR), HR status (ie, combining ER with PR), HER2, molecular subtype (HR+/HER2-, HR+/HER2+, HR-/HER2+, TNBC, or unknown), surgery (mastectomy or lumpectomy), and radiotherapy (yes or no/unknown).

### Statistical analysis

We plotted the cumulative mortality rates by causes of death (eg, death from breast cancer, other cancers and non-cancer) in NACT and ACT patients from breast cancer diagnosis to 10 years survivorship, respectively. We compared clinical characteristics between patients with NACT and ACT by logistic regression controlling for demographic characteristics (eg, age, year at diagnosis, race, cohabitation status, and percentile of cost of living and county) and tumor characteristics (eg, tumor stage, histology, tumor grade, ER status, PR status, and HER2 status). We also estimated the hazard ratios (HRs) and 95% confidence intervals (CIs) of breast cancer specific and overall mortality by comparing patients in NACT group with ACT group using Cox regression model. Taking account of the different risks of mortality in subgroups, we conducted 3 primary sets of survival analyses as below: (1) All NACT vs ACT patients—Cox proportional hazards regression models were fitted to estimate HRs and 95% CIs for breast cancer-specific mortality and overall mortality, with ACT as the reference. Adjustment was performed sequentially. Model A: demographic factors (age at diagnosis, year of diagnosis, race, cohabitation status, cost of living, county type). Model B: Model A + tumor characteristics (stage, histology, grade, ER, PR, HER2 status). Model C: Model B + treatment factors (surgery, radiotherapy). (2) NACT with pCR vs ACT—The same sequential modeling strategy was applied to the subgroup of NACT patients who achieved pCR, comparing their survival outcomes to ACT patients. (3) NACT with response other than pCR vs ACT—We analyzed separately (a) NACT patients with non-pCR and (b) NACT patients with documented response but unknown whether complete or partial, each compared with ACT patients, using the same sequentially adjusted Cox models as above.

To further explore the impact of clinical characteristics on breast cancer-specific mortality among NACT patients, we conducted analyses stratified by tumor and treatment features with the adjustment of model C. Since the decreased risk of breast cancer-specific mortality was detected in patients with pCR after NACT compared with ACT patients, we only ­performed subsequent analysis in this subgroup. We defined “NACT-disfavored subgroups” as strata where the fully adjusted Cox model (Model C) yielded an HR for NACT vs ACT >1 with a 95% CI excluding 1.

All statistical analyses were performed using STATA (version 16.0; Stata Corporation). *P* < .05 was considered as the statistical significance.

## Results

### Demographic and clinical characteristics

Compared with ACT patients, those in the NACT group were more likely to be diagnosed in recent years (2018-2021) and at a younger age (Table 1). Besides, NACT patients presented with more unfavorable tumor characteristics (ie, poorer differentiation, more advanced anatomic cancer stage, and a higher proportion of TNBC and HER2+ subtypes) (Table 2). Moreover, patients in the NACT group were more likely to undergo mastectomy and receive radiotherapy (Table 2).

**Table 1.** Baseline characteristics of breast cancer patients with neoadjuvant and adjuvant chemotherapy: a SEER population-based study in the United States, 2010-2021.

	ACT		NACT	
	*N*	%	*N*	%
**Total number**	124 202	-	48 350	-
**Year of diagnosis**				
**2010-2013**	49 238	39.6	9762	20.2
**2014-2017**	39 929	32.1	15 936	33.0
**2018-2021**	35 035	28.2	22 652	46.9
**Age at diagnosis (mean±SD), years**	55.7 ± 11.6		52.5 ± 12.3	
** Race **				
**White**	93 605	75.4	34 595	71.6
**Black**	15 988	12.9	7510	15.5
**Asian**	13 954	11.2	5897	12.2
**Other**	655	0.5	348	0.7
** Cohabitation status **				
**Non-cohabitation**	43 076	34.7	18 248	37.7
**Cohabitation**	76 649	61.7	28 558	59.1
**Unknown**	4477	3.6	1544	3.2
**County**				
**Counties in metropolitan areas of larger than 1 million population**	73 872	59.5	30 033	62.1
**Counties in metropolitan areas of 250 000 to 1 million population**	28 068	22.6	10 776	22.3
**Counties in metropolitan areas of less than 250 thousand population**	9326	7.5	3279	6.8
**Nonmetropolitan counties not adjacent to a metropolitan area**	5261	4.2	1673	3.5
**Nonmetropolitan counties adjacent to a metropolitan area**	7675	6.2	2589	5.4
** Cost of living adjusted median household income in the county of residence **				
**Lowest tertile**	20 844	16.8	6806	14.1
**Middle tertile**	35 085	28.2	13 021	26.9
**Highest tertile**	68 273	55.0	28 523	59.0

Abbreviations: ACT, adjuvant chemotherapy; N, number; NACT, neoadjuvant therapy; SD, standard deviation.

**Table 2.** Associations of clinical characteristics with neoadjuvant chemotherapy: a SEER population-based study in the United States, 2010-2021.

	ACT	NACT	
	*N* (%)	*N* (%)	OR (95% CI)^a^
**Histology**			
**Ductal**	101 064 (81.4)	42 414 (87.7)	1.00
**Lobular**	9711 (7.8)	2200 (4.6)	0.60 (0.57-0.63)
**Mixed**	9255 (7.5)	1978 (4.1)	0.59 (0.56-0.62)
**Others**	4172 (3.4)	1758 (3.6)	1.16 (1.09-1.23)
**Tumor grade**			
**Well differentiated**	9617 (7.7)	1964 (4.1)	1.00
**Moderately differentiated**	47 936 (38.6)	15 460 (32)	1.43 (1.36-1.51)
**Poorly/un differentiated**	61 824 (49.8)	28 256 (58.4)	1.98 (1.88-2.09)
**TNM Stage**			
**I**	52 128 (42)	10 567 (21.9)	1.00
**II**	53 778 (43.3)	22 969 (47.5)	3.27 (3.18-3.37)
**III**	18 296 (14.7)	14 814 (30.6)	6.34 (6.13-6.55)
**Molecular subtypes**			
**HR+/HER2-**	70 930 (57.1)	17 540 (36.3)	1.00
**HR+/HER2+**	21 235 (17.1)	11 518 (23.8)	2.21 (2.14-2.27)
**HR-/HER2+**	7769 (6.3)	5735 (11.9)	3.22 (3.09-3.35)
**Triple negative**	21 406 (17.2)	12 776 (26.4)	2.52 (2.45-2.60)

	*N* (%)	*N* (%)	OR (95% CI)^b^
**Surgery**			
**Lumpectomy**	65 083 (52.4)	18 778 (38.8)	1.00
**Mastectomy**	59 119 (47.6)	29 572 (61.2)	1.33 (1.30-1.37)
**Radiotherapy**			
**No/unknown**	47 675 (38.4)	15 687 (32.4)	1.00
**Yes**	76 527 (61.6)	32 663 (67.6)	1.20 (1.17-1.23)

Patients with missing information on grade (*N* = 7495,4.3%), or molecular types (*N* = 3643, 2.1%) were not included in the corresponding analysis.

Abbreviations: ACT, adjuvant chemotherapy; CI, confidence interval; HR, hormone-receptor; HER2, human epidermal growth factor receptor 2; N, number; NACT, neoadjuvant therapy; OR, odds ratio.

a The models were adjusted for age (continuous) and year at diagnosis, race, cohabitation status, percentile of cost of living and county.

b The models were additionally adjusted for tumor stage, histology, tumor grade, ER status, PR status, and HER2 status.

### Cumulative mortality rates between patients with NACT and ACT

**Figure 1.**
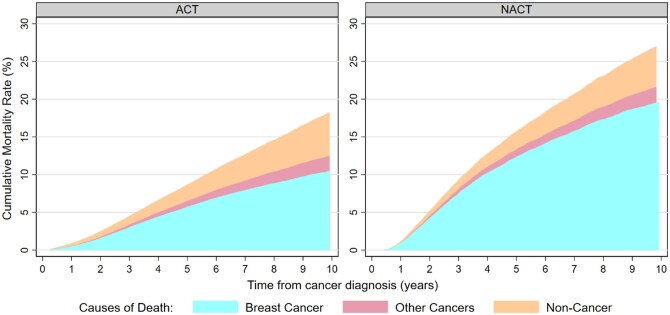
Cumulative mortality rates of deaths among breast cancer patients after neoadjuvant and adjuvant chemotherapy from cancer diagnosis to 10 years.

A total of 13 834 ACT and 6310 NACT patients died during the follow up. Compared with ACT patients, those in the NACT group had a higher cumulative mortality rate of breast cancer (19.60% vs 10.46%), but similar cumulative mortality rate of other cancers (2.00% vs 2.01%) and non-cancer (5.42% vs 5.84%), within 10 years after the initial breast cancer diagnosis (Figure 1). Nevertheless, among NACT patients who achieved pCR, 865 deaths were identified during the follow-up period, and these patients had significantly lower cumulative mortality rates for breast cancer (8.74%), other cancers (1.27%), and non-cancer causes (3.30%) (Figure S2).

### Overall and breast cancer-specific mortality risks in patients after NACT and ACT

The median [interquartile range] of follow-up was 69 [34, 105] and 41 [19, 75] months for ACT and NACT patients, respectively. A total of 8257 and 4800 breast cancer-specific deaths were identified in the 2 groups. Compared with ACT individuals, those in the NACT group were associated with an increased risk of breast cancer-specific mortality (HR 2.20, 95% CI 2.12-2.28), only adjusting for demographic characteristics. When additionally accounting for tumor characteristics, the elevated risk remained significant (HR 1.48, 95% CI 1.42-1.54). With further adjustment for treatment, a similar pattern was observed (HR 1.47, 95% CI 1.41-1.53). Moreover, NACT patients were at higher risk of overall mortality (HR 1.36, 95% CI 1.32-1.41). Stronger risks of breast cancer-specific and overall mortality were observed among NACT patients with non-pCR and those who responded to NACT but not noted if partial or complete response (Table 3). In contrast, NACT patients who achieved pCR had significantly reduced risks of both overall mortality (HR 0.63, 95% CI 0.58-0.67) and breast cancer–specific mortality (HR 0.59, 95% CI 0.54-0.64) compared with ACT patients (Table 3).

**Table 3.** Hazard ratios (HRs) of breast cancer-specific and overall mortality in patients receiving neoadjuvant chemotherapy, compared to adjuvant chemotherapy: a SEER population-based study in the United States, 2010-2021.

	ACT N (MR)	NACT N (MR)	HR (95% CI)^a^	HR (95% CI)^b^	HR (95% CI)^c^
**All patients in NACT group**					
**Breast cancer-specific mortality**	8257 (1.1)	4800 (2.4)	2.20 (2.12-2.28)	1.48 (1.42-1.54)	1.47 (1.41-1.53)
**Overall mortality**	13 834 (1.9)	6310 (3.1)	1.88 (1.83-1.94)	1.36 (1.32-1.41)	1.36 (1.32-1.41)
**pCR after NACT**					
**Breast cancer-specific mortality**	8257 (1.1)	589 (0.9)	0.87 (0.80-0.95)	0.59 (0.54-0.64)	0.59 (0.54-0.64)
**Overall mortality**	13 834 (1.9)	865 (1.3)	0.86 (0.80-0.92)	0.62 (0.58-0.67)	0.63 (0.58-0.67)
**Non-pCR after NACT**					
**Breast cancer-specific mortality**	8257 (1.1)	2838 (3.4)	3.11 (2.98-3.25)	1.98 (1.89-2.07)	1.97 (1.88-2.06)
**Overall mortality**	13 834 (1.9)	3643 (4.4)	2.56 (2.47-2.65)	1.79 (1.72-1.86)	1.79 (1.72-1.86)
**Response to NACT, but not noted if partial or complete response**					
**Breast cancer-specific mortality**	8257 (1.1)	1373 (2.6)	2.41 (2.27-2.55)	1.62 (1.52-1.72)	1.62 (1.53-1.72)
**Overall mortality**	13 834 (1.9)	1802 (3.5)	2.09 (1.99-2.20)	1.52 (1.44-1.60)	1.53 (1.46-1.61)

Abbreviations: ACT, adjuvant chemotherapy; NACT, neoadjuvant therapy; CI, confidence interval; HR, hazards ratio; MR, mortality rate per 100 person-years; N, number of deaths; pCR, pathological complete response.

a HR was adjusted for age (continuous) and year at diagnosis, race, cohabitation status, percentile of cost of living and county.

b HR was additionally adjusted for tumor stage, histology, grade, estrogen receptor status, progesterone receptor status, and human epidermal growth factor receptor 2 status.

c HR was additionally adjusted for surgery and radiotherapy.

### Breast cancer-specific mortality risk by clinical characteristics

**Figure 2.**
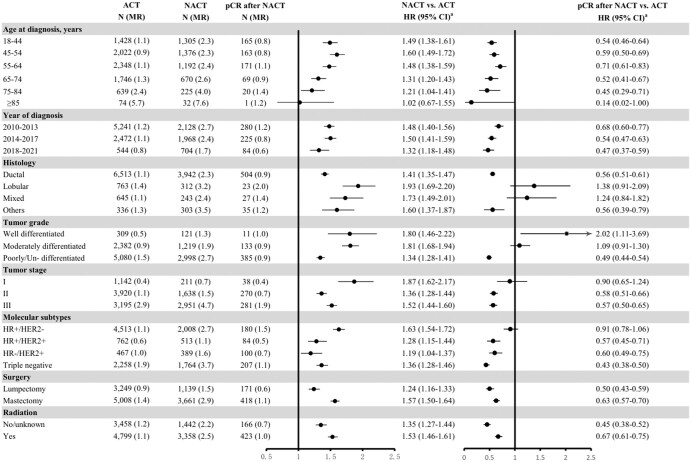
Hazard ratios (HRs) of breast cancer specific deaths amongst patients after NACT and patients with pCR after NACT by clinical characteristics, compared with the ACT patients: a population-based study in the United States, 2010-2021. ACT, adjuvant chemotherapy; NACT, neoadjuvant therapy; pCR, pathological complete response; CI, confidence interval; HR+, hormone-receptor positive, HR-, hormone-receptor negative; HER2, human epidermal growth factor receptor 2; HR, hazard ratio; MR, mortality rate per 100 person-years; N, number of deaths. ^a^HR was adjusted for age (continuous) and year at diagnosis, race, cohabitation status, percentile of cost of living and county, tumor stage, histology, tumor grade, estrogen receptor status, progesterone receptor status, and human epidermal growth factor receptor 2 status, surgery and radio-therapy.

When comparing NACT with ACT across clinical characteristics, a stronger association with breast cancer-specific mortality, independent of demographic factors, tumor characteristics and treatment modes, was observed for patients in NACT-disfavored subgroups including lobular/mixed histology, well/moderated differentiated tumor, stage I, HR+/HER2- molecular type (HR 1.63-1.93; *P* for interaction < .001; Figure 2). Additional analyses showed that patients who achieved pCR after NACT experienced improved breast cancer-specific survival compared with those in the ACT group, whereas NACT patients with pCR within NACT-disfavored subgroups did not experience a survival benefit (Figure 2).

## Discussion

This large population-based cohort study provides a comprehensive comparison of survival outcomes between early-stage breast cancer patients receiving NACT and ACT. Our findings reveal that patients treated with NACT—who typically present with more aggressive tumor biology and are more likely to undergo mastectomy and radiotherapy—exhibit worse breast cancer-specific survival compared with those treated with ACT. The elevated risk was particularly pronounced among patients diagnosed at perimenopausal age (45-54 years), those with lobular histology, well- or moderately differentiated tumors, local-stage disease, and the HR+/HER2- subtype. However, patients who achieved pCR after NACT demonstrated significantly improved survival outcomes, even surpassing those of patients treated with ACT.

The advent of NACT has provided new opportunities to assess treatment response before surgery, thus enabling the tailoring of subsequent therapies.^2^^,^^24^ We have summarized the largest and most impactful studies comparing NACT and ACT in Table S2. These landmark trials and meta-analyses consistently demonstrate that while NACT does not improve survival compared with ACT, pCR serves as a strong surrogate for long-term benefit, particularly in TNBC and HER2+ subgroups. Nevertheless, controversies remain regarding its role in NACT-disfavored subgroups, and post-NACT escalation strategies are under continuous investigation. Our study demonstrates that NACT patients have a significantly higher breast cancer-specific mortality rate (19.60% vs 10.46%) compared to ACT patients. NACT patients are associated with increased risk of breast cancer-specific mortality and overall mortality independent of demographic, tumor, and treatment-related factors, compared to individuals treated with ACT.

These findings align with previous research suggesting that NACT does not improve survival compared to ACT unless pCR is achieved.^5^^,^^7^ One possible explanation is selection bias, as NACT is often recommended for patients with more aggressive tumor phenotypes (eg, TNBC and HER2+) and advanced clinical stage at diagnosis.^9^ Indeed, our study confirms that NACT patients had a higher prevalence of TNBC, HER2+, and poorly differentiated tumors, leading to a worse baseline prognosis. Besides, NACT patients were more likely to receive mastectomy (61.2% vs 47.6%) and radiotherapy (67.6% vs 61.6%), suggesting a higher initial tumor burden requiring more aggressive local treatment.

A key finding of our study is that NACT patients achieving pCR had better survival outcomes compared to their ACT counterparts with death from any cause being more common, suggesting a significant clinical benefit for ones with pCR. Specifically, pCR after NACT was associated with a 41% lower breast cancer-specific mortality risk (HR 0.59, 95% CI 0.54-0.64) and a 37% lower overall mortality risk (HR 0.63, 95% CI 0.58-0.67) compared to ACT. These results reinforce the strong prognostic value of pCR, which has been well-documented in previous studies.^9^^,^^10^ Patients achieving pCR, particularly in TNBC and HER2+ subtypes, have significantly improved long-term survival, likely due to chemosensitivity and elimination of residual tumor burden.^6^^,^^10^^,^^11^^,^^25^^,^^26^ Conversely, non-pCR patients show persistent residual disease, which has been associated with increased recurrence risk and poorer survival outcomes.^27^^,^^28^ Therefore, there is no doubt that this underscores the need for post-NACT risk stratification, which might guide extra adjuvant therapy for non-pCR patients.^14^^,^^15^ Our stratified analysis further identifies specific NACT patient subgroups, including those with lobular/mixed histology and less aggressive tumor biology (eg, HR+/HER2−, well/moderately differentiated tumors, stage I), who exhibited greater mortality risk compared to ACT patients.

Our findings underscore that the benefit of NACT is biology- and response-dependent. Breast cancer patients with pCR—most commonly TNBC and HER2+ subtypes—experienced favorable survival outcomes, supporting continued use of NACT where chemosensitivity and pCR rates are high. For non-pCR after NACT, evidence-based post-neoadjuvant escalation (eg, capecitabine in TNBC, T-DM1 in HER2+ disease, PARP inhibition for germline BRCA1/2, and CDK4/6 inhibition for high-risk HR+/HER2−) remains central to risk reduction.^19^ In contrast, patients with HR+/HER2− disease, lobular or mixed histology, well/moderately differentiated tumors, and stage I disease derived limited survival benefit from NACT in our cohort, even when pCR was achieved in subgroup analyses, indicating NACT should be reconsidered in these populations. Moreover, for HR+/HER2− tumors—particularly lobular/mixed histology, low-grade phenotype—up-front surgery with genomic risk–adapted adjuvant therapy or neoadjuvant endocrine therapy (NET) with on-treatment response assessment may represent more appropriate initial strategies than NACT. Emerging antibody–drug conjugates and biomarker-driven approaches may further refine outcomes in low-chemosensitivity phenotypes and warrant focused investigation in the neoadjuvant and post-neoadjuvant settings.^29^

By leveraging a large, contemporary, population-based cohort, our analysis demonstrates that the effect of NACT varies across biologic subtypes and grades, with concentrated benefit linked to pCR and limited value in HR+/HER2−, lobular/mixed, well-/moderately differentiated, and stage I subgroups. These data support biomarker- and response-guided sequencing—including surgery-first or NET—to better align treatment with tumor biology.

Our findings highlight the importance of personalized treatment strategies for breast cancer patients, particularly when considering molecular subtypes and response to neoadjuvant therapies. However, it is essential to recognize that the standard of care in breast cancer treatment is constantly evolving, and new therapies are likely to influence both clinical decision-making and survival outcomes in future analyses. For example, in the United States, neoadjuvant treatments like pembrolizumab (Keynote-522),^30^ which was not a standard of care in 2021, have been integrated into treatment regimens for triple-negative breast cancer (TNBC) based on promising clinical trial results. This shift toward incorporating immune checkpoint inhibitors in neoadjuvant settings for high-risk patients, particularly those with TNBC, could undoubtedly impact future treatment strategies, further improving survival outcomes. As the field continues to advance with the introduction of immunotherapy and other novel agents, subsequent studies will need to account for these changes in treatment paradigms to provide insights into longer-term survival and better therapeutic effectiveness.

The main strength of this study is the large population cohort of breast cancer patients, ensuring minimalized selection biases. One of the major concerns is the lack of some baseline factors (eg, comorbidities^31^, performance status,^32^ and body mass index^33^) and detailed treatment data (eg, specific chemotherapy regimens, endocrine therapy, or targeted therapy details), which may influence survival outcomes. However, these factors are affected by treatment modes (eg, consideration of the patient’s general physical condition in treatment modality selection) and tumor characteristics (eg, molecular subtype as a determinant of endocrine therapy and targeted therapy), which have been elaboratively controlled in our analyses. Thus, we attempted to reduce confounding from these unmeasured factors by adjusting for demographic, tumor, and treatment variables that are correlated with them, but residual confounding cannot be entirely excluded. Second, NACT patients differed from ACT individuals regarding age and clinical characteristics. Nevertheless, we have conducted stratified analyses by age at diagnosis (eg, Figure 2) and tumor factors (eg, Figure 2) where patients between groups were highly comparable. Third, one notable limitation of this study is the relatively short median follow-up period of 5 years, which may be insufficient to fully capture the long-term survival outcomes, especially for HR+ breast cancer patients. While 5 years of follow-up is typical in many breast cancer studies, HR+ tumors are known to have a slower recurrence pattern, and longer follow-up is essential to better understand the long-term survival trends, particularly for patients with less aggressive tumor types. Further studies with extended follow-up are needed to assess the durability of the survival benefits observed in this cohort. Fourth, the absolute number of patients with lobular or mixed histology and well-differentiated tumors was relatively small in the NACT group compared with the ACT group. As given in Table 2, lobular histology accounted for 4.6% of NACT patients vs 7.8% of ACT patients, and mixed histology for 4.1% vs 7.5%, respectively. Well-differentiated tumors represented 4.1% in the NACT group vs 7.7% in the ACT group, while moderately differentiated tumors accounted for 32.0% vs 38.6%. For stage I disease, the proportion was 21.9% in NACT vs 42.0% in ACT, confirming that most NACT patients presented with higher stage and/or more advanced nodal status at baseline. We acknowledge that the relatively small sample sizes within some of these emphasized subgroups—particularly lobular/mixed histology and well-differentiated tumors—may have contributed to wider confidence intervals and should be interpreted with caution. Nevertheless, the consistent direction and magnitude of risk differences across analyses suggest that the observed associations are unlikely to be solely due to chance, and they merit further validation in larger datasets.

## Conclusion

NACT patients have worse survival, compared with their ACT counterparts. Although patients with pCR after NACT benefit from survival outcomes, NACT-disfavored subgroups may derive limited benefit from NACT, and alternative approaches should be considered.

## Supplementary Material

oyaf356_Supplementary_Data

## Data Availability

The datasets used and/or analyzed during the current study are available from the corresponding author on reasonable request.
