# Supplementary material for: Survival outcomes for neoadjuvant versus adjuvant chemotherapy in early breast cancer patients
Source: Oncologist. 2025 Nov 18;30(11):oyaf356. doi: 10.1093/oncolo/oyaf356 (PMC12623009; doi:10.1093/oncolo/oyaf356)
Supplement: oyaf356_Supplementary_Data [file oyaf356_supplementary_data.zip › TableS1.docx]

| **Table S1. Baseline characteristics of NACT patients with pCR, non-pCR and** **response to NACT but not noted if partial or complete response: a SEER population-based study in US, 2010-2021.** | | | | | | |
| --- | --- | --- | --- | --- | --- | --- |
|  | **pCR after NACT** | | **Non-pCR after NACT** | | **Response to NACT but not noted if partial or complete response** | |
|  | **N** | **%** | **N** | **%** | **N** | **%** |
| **Total number** | 15,525 | - | 19,998 | - | 12,827 | - |
| **Year of diagnosis** |  |  |  |  |  |  |
| 2010-2013 | 2,710 | 17.5 | 4,843 | 24.2 | 2,209 | 17.2 |
| 2014-2017 | 5,076 | 32.7 | 6,160 | 30.8 | 4,700 | 36.6 |
| 2018-2021 | 7,739 | 49.8 | 8,995 | 45.0 | 5,918 | 46.1 |
| **Age at diagnosis (mean±SD), years** | 51.5±12.1 | | 55.7±11.6 | | 52.7±12.4 | |
| **Race** |  |  |  |  |  |  |
| White | 11,334 | 73.0 | 14,237 | 71.2 | 9,024 | 70.4 |
| Black | 2,180 | 14.0 | 3,095 | 15.5 | 2,235 | 17.4 |
| Asian | 1,895 | 12.2 | 2,520 | 12.6 | 1,482 | 11.6 |
| Other | 116 | 0.7 | 146 | 0.7 | 86 | 0.7 |
| **Cohabitation status** |  |  |  |  |  |  |
| Non-cohabitation | 5,431 | 35.0 | 7,945 | 39.7 | 4,872 | 38.0 |
| Cohabitation | 9,623 | 62.0 | 11,410 | 57.1 | 7,525 | 58.7 |
| Unknown | 471 | 3.0 | 643 | 3.2 | 430 | 3.4 |
| **County** |  |  |  |  |  |  |
| Counties in metropolitan areas of larger than 1 million population | 9,690 | 62.4 | 12,409 | 62.1 | 7,934 | 61.9 |
| Counties in metropolitan areas of 250,000 to 1 million population | 3,536 | 22.8 | 4,602 | 23.0 | 2,638 | 20.6 |
| Counties in metropolitan areas of less than 250 thousand population | 1,014 | 6.5 | 1,319 | 6.6 | 946 | 7.4 |
| Nonmetropolitan counties not adjacent to a metropolitan area | 478 | 3.1 | 658 | 3.3 | 537 | 4.2 |
| Nonmetropolitan counties adjacent to a metropolitan area | 807 | 5.2 | 1,010 | 5.1 | 772 | 6.0 |
| **Cost of living adjusted median household income in the county of residence** |  |  |  |  |  |  |
| Lowest tertile | 1,957 | 12.6 | 2,782 | 13.9 | 2,067 | 16.1 |
| Middle tertile | 4,037 | 26.0 | 5,544 | 27.7 | 3,440 | 26.8 |
| Highest tertile | 9,531 | 61.4 | 11,672 | 58.4 | 7,320 | 57.1 |
| **Histology** |  |  |  |  |  |  |
| Ductal | 14,362 | 92.5 | 16,871 | 84.4 | 11,181 | 87.2 |
| Lobular | 279 | 1.8 | 1,276 | 6.4 | 645 | 5.0 |
| Mixed | 357 | 2.3 | 1,046 | 5.2 | 575 | 4.5 |
| Others | 527 | 3.4 | 805 | 4.0 | 426 | 3.3 |
| **Tumor grade** |  |  |  |  |  |  |
| Well differentiated | 253 | 1.6 | 1,104 | 5.5 | 607 | 4.7 |
| Moderately differentiated | 3,561 | 22.9 | 7,368 | 36.8 | 4,531 | 35.3 |
| Poorly/Un differentiated | 11,004 | 70.9 | 10,317 | 51.6 | 6,935 | 54.1 |
| Unknown | 707 | 4.6 | 1,209 | 6.0 | 754 | 5.9 |
| **TNM Stage** |  |  |  |  |  |  |
| I | 3,312 | 21.3 | 4,220 | 21.1 | 3,035 | 23.7 |
| II | 8,634 | 55.6 | 8,541 | 42.7 | 5,794 | 45.2 |
| III | 3,579 | 23.1 | 7,237 | 36.2 | 3,998 | 31.2 |
| **Molecular subtypes** |  |  |  |  |  |  |
| HR+/HER2- | 2,694 | 17.4 | 9,530 | 47.7 | 5,316 | 41.4 |
| HR+/HER2+ | 4,421 | 28.5 | 4,029 | 20.1 | 3,068 | 23.9 |
| HR-/HER2+ | 3,305 | 21.3 | 1,298 | 6.5 | 1,132 | 8.8 |
| Triple negative | 4,894 | 31.5 | 4,781 | 23.9 | 3,101 | 24.2 |
| Unknown | 211 | 1.4 | 360 | 1.8 | 210 | 1.6 |
| **Surgery** |  |  |  |  |  |  |
| Lumpectomy | 6,885 | 44.3 | 7,043 | 35.2 | 4,850 | 37.8 |
| Mastectomy | 8,640 | 55.7 | 12,955 | 64.8 | 7,977 | 62.2 |
| **Radiotherapy** |  |  |  |  |  |  |
| No/unknown | 5,815 | 37.5 | 6,037 | 30.2 | 3,835 | 29.9 |
| Yes | 9,710 | 62.5 | 13,961 | 69.8 | 8,992 | 70.1 |
| Abbreviations: NACT, neoadjuvant therapy; pCR, pathological complete response; HR, hormone receptor; HER2, human epidermal receptor 2; N, number; SD, standard deviation. | | | | | | |
