# Supplementary material for: Survival outcomes for neoadjuvant versus adjuvant chemotherapy in early breast cancer patients
Source: Oncologist. 2025 Nov 18;30(11):oyaf356. doi: 10.1093/oncolo/oyaf356 (PMC12623009; doi:10.1093/oncolo/oyaf356)
Supplement: oyaf356_Supplementary_Data [file oyaf356_supplementary_data.zip › TableS2_NACT_vs_ACT.docx]

| Table S2. Key studies comparing survival outcomes between neoadjuvant (NACT) and adjuvant chemotherapy (ACT) in early breast cancer | | | | | | |
| --- | --- | --- | --- | --- | --- | --- |
| Study / Year | Population size | Subtype | Intervention | Main outcomes | Major findings | Journal |
| EBCTCG Meta-analysis, 2018 | 10 RCTs, 4756 pts, stage I–III | HR+ and HR- | NACT vs. ACT  Most chemotherapy was anthracycline based | OS, BCSS,DFS, local and distant recurrence | No OS difference; NACT ↑ breast conservation but ↑ local recurrence. | Lancet Oncol |
| CTNeoBC pooled analysis, 2014 | 12 trials, 11,955 pts | HR+/HER2-;HR+/HER2+;HR-/HER2+;TNBC | NACT | pCR vs. survival | pCR is associated with improved EFS & OS, esp. in TNBC & HER2+ | Lancet |
| NSABP B-18 & B-27, 2008 | 1,523 pts in B18 ;  2,411 pts in B27 | - | Pre-op vs. post-op chemo | OS, DFS, pCR | No OS/DFS difference; pCR = surrogate of benefit.  NACT ↑ local recurrence.  T added to AC regimen in NACT ↑ pCRs | J Clin Oncol |
| NeoSphere, 2016 | 417 pts | HER2+ | NACT + pertuzumab and trastuzumab | pCR, PFS, OS | Dual HER2 blockade ↑ pCR; trend to ↑ survival  pCR could be an early indicator of survival outcome | Lancet Oncol |
| CALGB 40603, 2022 | 443 pts | TNBC | NACT ± carboplatin/bevacizumab | pCR, EFS, OS | Carboplatin ↑ pCR but no DFS or OS improvement | J Clin Oncol |
| Keynote-522, 2020/2024 update | 1,174 pts | TNBC | NACT ± pembrolizumab | pCR, EFS, OS | Pembrolizumab ↑ pCR & OS benefit | NEJM |
| KATHERINE, 2019/2023 update | 1,486 pts with residual disease | HER2+ | Post-NACT: T-DM1 vs. trastuzumab | IDFS, OS | T-DM1 significantly ↑ IDFS & OS | NEJM |
| CREATE-X, 2017 | 910 with residual disease | HER2- | Post-NACT: capecitabine vs. none | DFS, OS | Capecitabine ↑ DFS & OS, esp. TNBC | NEJM |
| monarchE, 2023 interim | 5,637 high-risk^a^ pts | HR+/HER2- | Post-NACT/ACT: abemaciclib + ET | IDFS | Abemaciclib ↑ IDFS | Lancet Oncol |
| OlympiA, 2021/2022 update | 1,836 pts | gBRCA1/2 mutated | Post-NACT/ACT: olaparib vs. placebo | DFS, OS | Olaparib ↑ DFS & OS | NEJM, Ann Oncol |
| Abbreviations: ACT, adjuvant chemotherapy; NACT, neoadjuvant chemotherapy; OS, overall survival; DFS, disease-free survival; BCSS, breast cancer-specific survival; EFS, event-free survival; IDFS, invasive disease-free survival; PFS, progression-free survival; pCR, pathological complete response; TNBC, triple-negative breast cancer; HER2, human epidermal growth factor receptor 2; ET, endocrine therapy; RCT, randomized controlled trial; pts, participants.  ^a^: High-risk disease was defined as either four or more positive axillary lymph nodes, or between one and three positive axillary lymph nodes and either grade 3 disease or tumour size of 5 cm or larger (cohort 1). A smaller group of patients were enrolled with between one and three positive axillary lymph nodes and Ki-67 of at least 20% as an additional risk feature (cohort 2). | | | | | | |
