# Supplementary figures and images for: Survival outcomes for neoadjuvant versus adjuvant chemotherapy in early breast cancer patients
Source: Oncologist. 2025 Nov 18;30(11):oyaf356. doi: 10.1093/oncolo/oyaf356 (PMC12623009; doi:10.1093/oncolo/oyaf356)

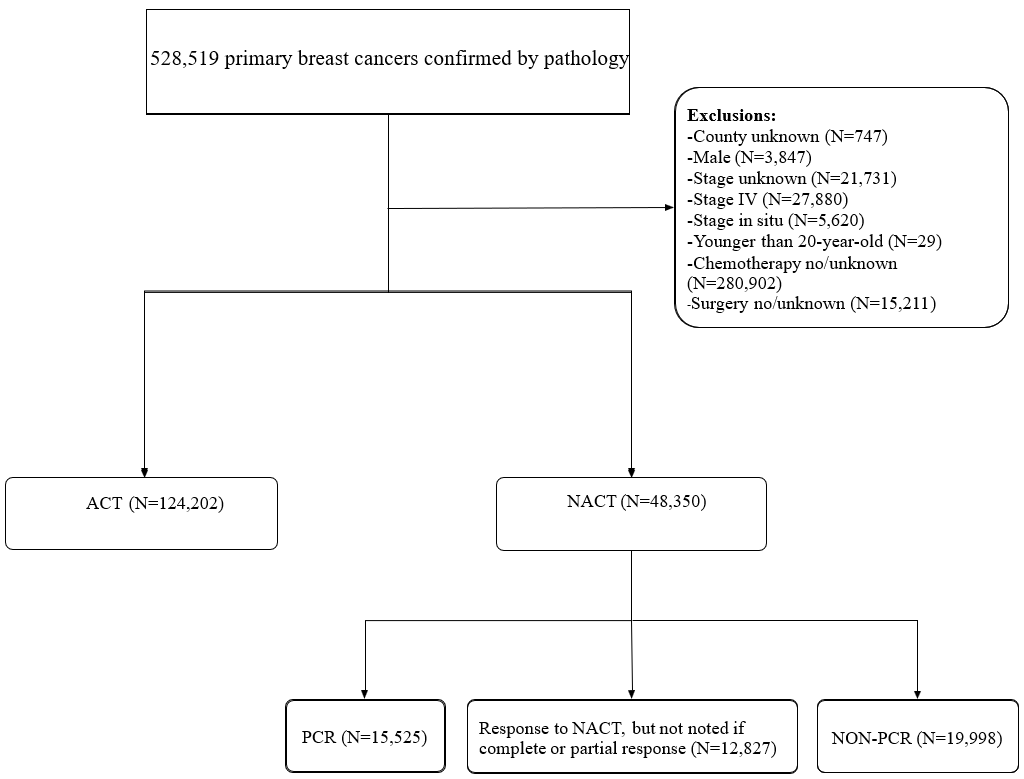

Supplement: oyaf356_Supplementary_Data [file oyaf356_supplementary_data.zip › Figure S1.tif]

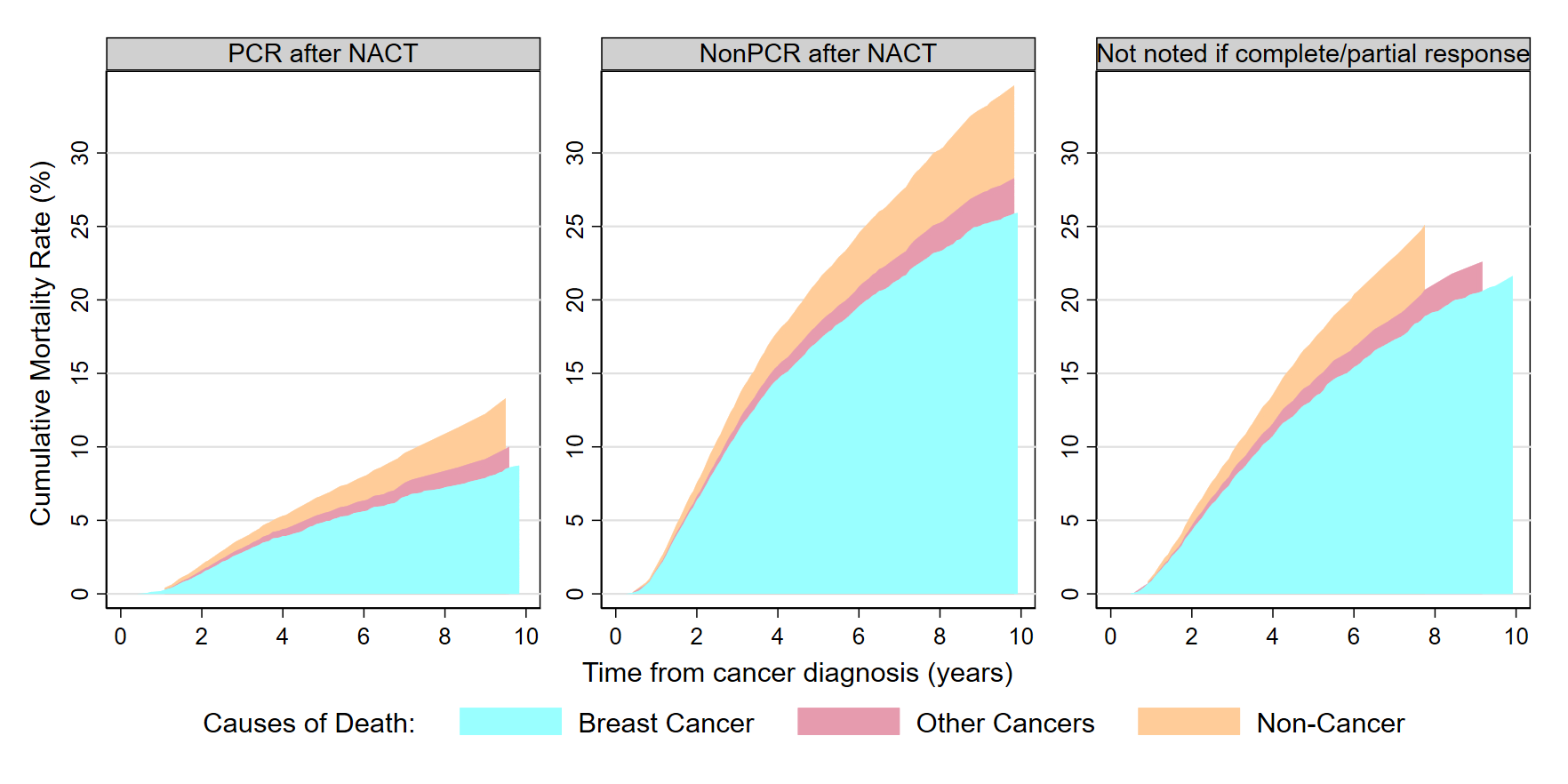

Supplement: oyaf356_Supplementary_Data [file oyaf356_supplementary_data.zip › Figure S2.tif]
